# Diffuse reflectance spectroscopy and imaging for non-invasive objective assessment of genitourinary syndrome of menopause: a pilot study

**DOI:** 10.1038/s41598-023-49655-4

**Published:** 2024-01-11

**Authors:** U. S. Dinish, Susan Logan, Ghayathri Balasundaram, Valerie Teo Xinhui, Keertana Vinod Ram, Zhang Ruochong, Bi Renzhe, Steffie Silvani, Kee Hua Cheng, Xu Xia, Goh Giap Hean, Mahesh Choolani, Malini Olivo

**Affiliations:** 1 Institute of Materials Research and Engineering (IMRE), Agency for Science, Technology and Research (A*STAR), 2 Fusionopolis Way, Innovis #08-03, Singapore, 138634 Republic of Singapore; 2 Department of Obstetrics and Gynaecology, NUS Yong Loo Lin School of Medicine, National University of Singapore, 1E Kent Ridge Road, NUHS Tower Block, Level 12, Singapore, 119228 Singapore; 3 Department of Obstetrics and Gynaecology, National University Hospital, 1E Kent Ridge Road, NUHS Tower Block, Level 12, Singapore, 119228 Singapore; 4 Department of Pathology, National University Hospital, 5 Lower Kent Ridge Road, Singapore, 119074 Singapore; 5 Present Address: A*STAR Skin Research Labs (A*SRL), Agency for Science, Technology and Research (A*STAR), 31 Biopolis Way, #07-01 Nanos, Singapore, 138669 Republic of Singapore; 6 Present Address: Department of Sexual and Reproductive Health, NHS Grampian, Aberdeen, Scotland UK

**Keywords:** Biophysics, Diseases, Engineering, Optics and photonics, Physics

## Abstract

The genitourinary symptom of menopause (GSM) affects up to 65% of women, resulting in symptoms such as vulvovaginal dryness, discomfort, and dysuria, which significantly impacts quality of life. The current assessment methods rely on subjective questionnaires that can be influenced by individual differences, as well as invasive measurements that are time-consuming and not easily accessible. In this study, we explore the potential of a non-invasive and objective assessment tool called diffuse reflectance spectroscopy and imaging (DRSI) to evaluate tissue chromophores, including water, lipid, oxyhemoglobin, and deoxyhemoglobin. These measurements provide information about moisture content, lipid levels, oxygen saturation, and blood fraction, which can serve as surrogate markers for genital estrogen levels. Our findings reveal distinct differences in these chromophores among pre, peri, and postmenopausal subjects. By using lipid and blood fraction tissue chromophores in a K-Nearest Neighbour classifier model, we achieved a prediction accuracy of 65% compared to vaginal maturation index (VMI) that is clinically used to assess estrogen-related hormonal changes. When age was included as the third feature, the accuracy increased to 78%. We believe that by refining the study protocol and configuring the fiber probe to examine tissue chromophores both in the superficial vulva skin for epidermal water content and the deeper layers, DRSI has the potential to provide objective diagnosis and aid in monitoring the treatment outcome of GSM.

## Introduction

Genitourinary syndrome of menopause (GSM) is a hypoestrogenism-induced condition characterized by various symptoms^1,2^. Pathophysiologically, GSM involves collagen fiber fusion, elastin fiber fragmentation, and loss of tissue elasticity. These changes lead to the disappearance of vaginal rugae, resulting in a shorter and narrower vaginal canal. Vascular support reduction contributes to decreased transudate volume and glandular secretions^3^. Thinning of the vaginal squamous epithelium causes friability, leading to petechiae, ulcerations, and bleeding from minor trauma^4–7^. Additionally, vaginal flora alterations, loss of lactobacilli, increased pH, and changes in the microbiome occur due to the thinning and decreased glycogenated superficial cells^6^, promoting the growth of pathogenic bacteria, vaginal discharge, and odour^8^. Secondary symptoms encompass genital dryness, vaginal burning, irritation, dyspareunia, as well as urinary urgency, dysuria, and recurrent urinary tract infections^9^. These symptoms are reported by over 50% of postmenopausal women and 15% of premenopausal women^1^. Due to its sensitive nature and preconceived notions, GSM is often underdiagnosed^10^, which may lead to underestimation of its prevalence.

Current GSM diagnosis and assessment involve subjective and objective methods. Subjective assessments include clinical interviews and pelvic examinations. Validated tools like most bothersome symptom (MBS), vulvar quality of life index (VQLI), vulva health index (VuHI), and vaginal health index (VHI) scoring systems are used to determine GSM severity. However, these subjective assessments may be biased due to variations in clinician criteria and personal judgement. Existing tools, therefore, may not adequately address biological and sociocultural differences among Asians, as most research has been conducted in Western populations^11^.

In contrast, vaginal pH, vaginal maturation index (VMI), and vulva/vaginal biopsy offer objective assessments of GSM. While vaginal pH remains the most inexpensive and rapid method, vaginal infections and intimate products may influence test results^12,13^. VMI quantifies the estrogen status of the vaginal epithelium. However, it is invasive, requires cytological expertise, and is costly to perform. Recent research has demonstrated histological and immunohistochemistry tests on vaginal biopsies to assess epithelial thickness, vascularization and treatment efficacy. Due to pain, potential complications, cost, and no malignant progression, biopsy is seldom used outside research^14^. Similarly, treatments, be they complementary, pharmaceutical or energy-based devices, experience similar issues when assessing efficacy. As such, we agree with Menison et al.^14^ when they state, “The literature on assessing GSM objectively is scant, and there is a need to develop an objective model for the most appropriate assessment for GSM”.

Optical spectroscopy is an efficient, non-invasive, and non-destructive analytical method that employs the principle of light reflectance to assess biochemical characteristics. In recent years, it has been increasingly utilized in healthcare for disease characterization, detection, treatment selection, and monitoring^15^. The intensity of reflected light at various wavelengths is dependent on the absorption and scattering of the body chromophores like water, lipid, oxyhaemoglobin (HbO_2_), and deoxyhaemoglobin (Hb). This allows for quantitative measurements of functional properties like moisture content, lipid, oxygen saturation (sO_2_), and blood fraction, which are metrics indicative of disease prognosis. In the field of dermatology, light has become an integral part of routine diagnostic practice by dermatologists^16^.

One subclass of optical spectroscopy is diffuse reflectance spectroscopy (DRS) which measures the diffused light from the region of interest (ROI). The modalities like fiber optic-based DRS, Raman spectroscopy, fluorescence imaging, optical coherence tomography, and visible-near infrared spectroscopy are already being used in the detection of cervical and ovarian cancer^17,18^. But the application of tissue optics pertaining to the symptoms of GSM is unexplored. This study is set out to examine the potential non-invasive usage of diffuse reflectance spectroscopy and imaging (DRSI) system to objectively estimate changes in vulva/vaginal skin health of pre and postmenopausal women. We hypothesize, in this pilot study, that the tissue chromophores obtained from DRS spectra can be surrogate markers of estrogen levels that are impacted in GSM. To the best of our knowledge, DRS has not been utilized for GSM assessment yet. The entire scope of this clinical study will cover technical details of the probe design and the data analysis approach followed by highlighting and discussing key results. The findings will generate new insights for the objective diagnosis of GSM, its treatment monitoring, and recommendations for future clinical research.

## Methods

### Patient recruitment criteria and demographics

All patients were recruited from the outpatient gynecology clinic at Singapore’s National University Hospital. A total of 100 participants with 50 premenopausal and 50 postmenopausal subjects were recruited. All subjects were Chinese females aged 21–69 years old, previously, or currently sexually active, willing to provide consent, and understand English. To minimize the confounding factors and enhance the internal validity, we intentionally restricted this study population to Chinese females. This deliberate choice was made to control the melanin-related variations and to ensure a more focused examination of our research objectives. Ethnicity of subjects was determined based on national records, utilizing self-reported information provided by participants during data collection. Exclusion criteria comprised the following: inability to undergo a speculum examination, application of vaginal estrogen, talcum powder, topical moisturizing gel or any kind of vaginal pharmaceutical treatment for GSM in the previous 72 h; use of systemic menopausal hormone therapy; dermatological conditions affecting the vulva; symptoms of abnormal vaginal discharge; pregnancy; breastfeeding and smoking. Participants provided written informed consent. All experiments were performed in accordance with relevant institutional guidelines and regulations. The protocol was approved by the Domain Specific Review Board of the National Healthcare Group, Singapore (Reference number 2020/01321).

### Clinical measurements

Demographics such as age, menopausal status, marital status, sexual activity in the past four weeks and clinical parameters such as height (m), weight (kg), and body mass index (BMI) (kg/m^2^) were collected. Women were classified as premenopausal if menstruated in the last one year and postmenopausal if amenorrhoeic for 12 consecutive months or more^19^. Premenopausal was further subdivided based on the frequency of menstrual cycles in the last 12 months into premenopausal (experienced no change in menstrual frequency in the past 12 months) and perimenopausal (experienced changes in the menstrual frequency or 3 to 11 months of amenorrhea) to capture the differences more accurately.

Participants completed an MBS questionnaire which comprised marking the severity of the following symptoms: vaginal dryness, vaginal (or vulva) irritation/burning/itching, dysuria, dyspareunia, and bleeding with sex^20^. Participants then completed the VQLI questionnaire, comprising seven domains and 15 questions^21^. The VQLI determines the extent of physical, psychosocial, and sexual aspects of vulva disease. Each symptom was scored 0–3. The overall score ranges from 0–5, 6–13, 14–23, 24–37 and 38–45, associating with minimal, mild, moderate, severe, and very severe, respectively^21^.

Participants were positioned in a prone, frog-legged posture for standardized visual examination of the external genitalia and vagina for VuHI and VHI, respectively. The VuHI evaluates vulvar components such as labia majora and minora, clitoris, introitus, elasticity, color, discomfort/pain, and other findings like petechiae, excoriation, and ulceration. Each component is ranked on a severity scale from normal (0) to severe (3), with a total score of > 8 suggesting severe vulva atrophy^22^. Following that, a speculum examination was conducted, and the VHI assessed overall elasticity, fluid secretion, epithelial mucosa, and moisture, which are subjective and objective pH measurements on a 5-point scale ranging from none to excellent. A total score ranging from 5 to 25 was calculated, with a score < 15 indicating vaginal atrophy^23^. Vaginal pH was measured using Whatman™ pH indicator paper (0–14 range) was placed against the lateral apical vaginal mucosa for five seconds, and the pH value was noted^24^. All the questionnaires filled by the patients and clinicians are documented in the supplementary information (Sect. 1). The VMI (Vaginal Maturation Index) was determined by obtaining a brush sample from the upper 1*/*3rd lateral vaginal wall and placing it in a standard BD SurePath™ Liquid-based Pap Test container^25^. Samples were processed using the PrepStain slide processor and standard protocols at the NUH cytology laboratory. The samples stained for cell visualization were read by two trained cytotechnologists, estimating the proportions of parabasal, intermediate, and superficial cells, based on a 100 cell-count representation. A predominance of parabasal cells and the absence of superficial cells indicate low estrogen levels. The cytology report was validated by a pathologist, and a smear was considered atrophic if the percentage of superficial cells was < 5%^26,27^. The VMI score was calculated using Eq. (1)^28,29^, where a score between 0 and 50 suggests low estrogen, while a score higher than or equal to 50 indicates normal estrogen^30^.

1 $${\text{VMI}}=(0\times P) +(1\times S) +(0.5\times I)$$

where P is the percentage of parabasal cells, S is the percentage of superficial cells, and I is the percentage of intermediate cells.

### Diffuse reflectance spectroscopy and imaging system

**Figure 1:**
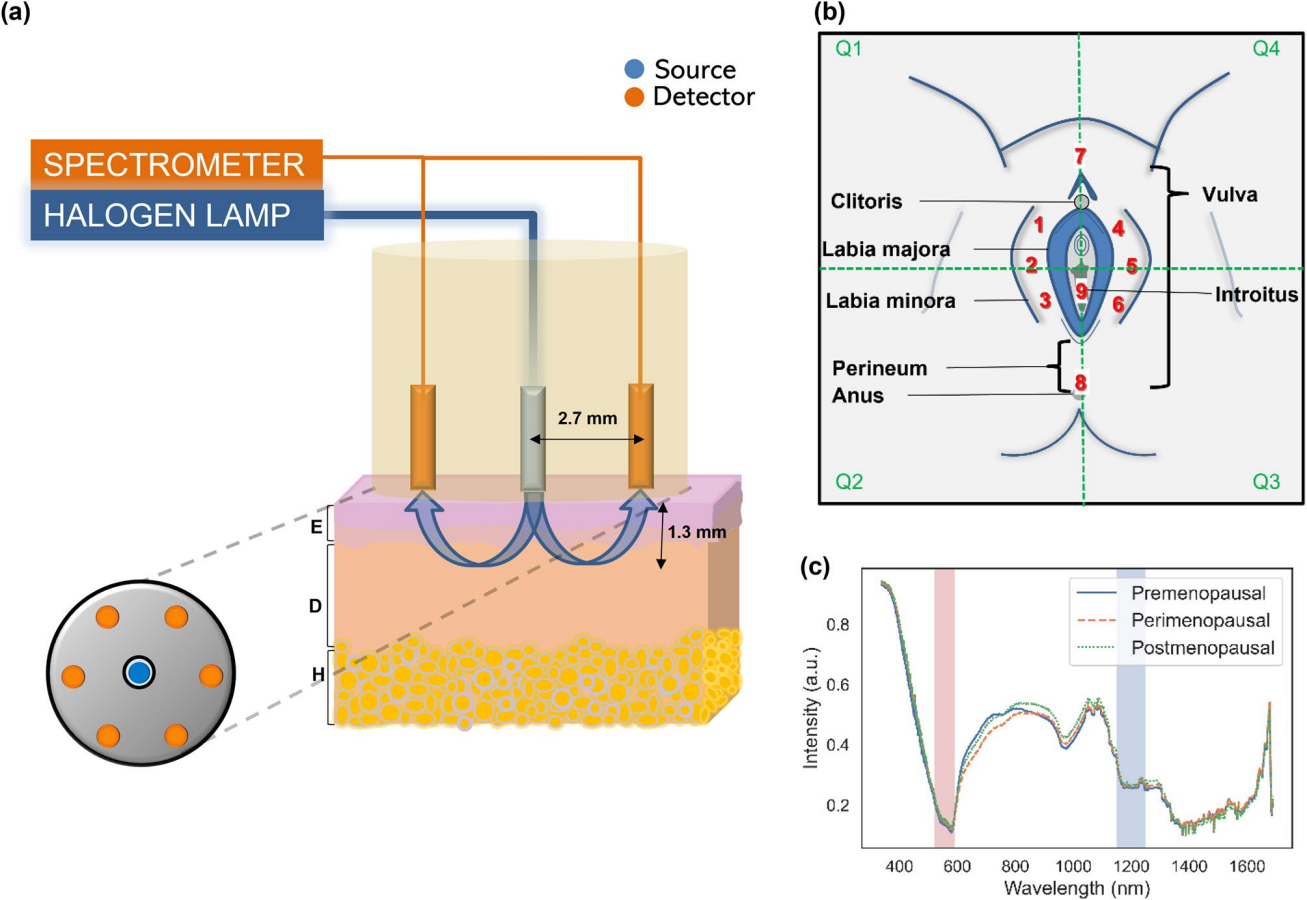
Diffuse reflectance spectroscopy and imaging (DRSI) system. (**a**) Illustration of probe parameters and detection depth in the vulvar skin (E: epidermis, D: dermis, H: hypodermis) in the transverse view. (**b**) Schematic of the investigated vulva locations marked from 1 to 9 which display GSM symptoms. (**c**) Raw spectrum of the average of all locations for all premenopausal (solid line), perimenopausal (dashed line), and postmenopausal (dotted line) subjects.

The DRSI system comprises a main console with two flexible handheld connections: 1. a custom fiber optic probe for vulva skin illumination and DRS measurements (Fig. 1a), and 2. a microscopy-based camera (USB Digital Microscope 2 M, RS PRO) for magnified images of the ROI. A 10 W Avalight-HAL tungsten halogen light source (AvaLight-HAL-S series, Avantes) with wavelengths ranging from 360 to 2500 nm illuminated the vulva skin through the central fiber of the probe. The power measured at the illumination fiber output is 4.5 mW. DRS spectral measurements were collected using a high sensitivity, low stray light, visible-near infrared spectrometer (AvaSpec-ULS2048XL-EVO) in the range of 360 to 1100 nm and a thermoelectrically cooled near-infrared spectrometer (AvaSpec-NIR512-1.7TEC) in the NIR range of 1000 to 1700 nm (InGaAs photodiode). The probe, as shown in Fig. 1a, consists of one source illumination multimode fiber (core diameter 600 µm) and six detector multimode fibers (core diameter 200 µm). The source-detector separation (SDS) was maintained at 2.7 mm which approximates a sampling depth (depth that 50% of the photons reach) of 1.3 mm^31,32^. The probe provided measurements of water, lipid, sO_2_, and blood fraction concentrations in the ROIs. It is a certified clinical research material (CRM2000221) approved by the Health Sciences Authority (HSA), Singapore. The calculation of negligible thermal load on vulva skin can be found in supplementary information (Sect. 2).

During the examination, the assessor initially positions the imaging camera towards the ROI, adjusts the focus, and captures images of the vulva region’s four quadrants (Fig. 1b). These images help identify any anatomical factors contributing to abnormal spectra. Subsequently, the clinician utilizes the DRS probe to capture spectra at nine different locations within the ROI (Fig. 1b). The entire process of capturing images and spectral measurements for each location takes less than 10 and 30 s, respectively, with the total acquisition time amounting to approximately 5 min per subject. After completing the readings, the probe is disinfected following standard protocols using biodegradable Wip’Anios Excel wipes. Finally, participants are asked to rate the discomfort level experienced during DRSI measurements on a scale of 1–5 (very uncomfortable to comfortable). Similarly, the clinician’s perceived difficulty in performing the DRSI measurements is assessed on a scale of 1–5 (very difficult to easily performed).

### Spectra and data processing

DRS employs the detection of tissue chromophores using tissue absorption and scattering model. In this study, we obtain four optical metrics namely water, lipid, HbO_2_, and Hb. Figure 1c represents the average raw spectrum for all locations in all premenopausal, perimenopausal, and postmenopausal subjects with the 520 to 590 nm region used for unmixing the sO_2_ and blood fraction components and the 1150 to 1250 nm region used for unmixing the water and lipid components.

The raw spectra S(*λ*_*i*_) collected for each location were divided by the white reflectance (5″ Spectralon^®^ Diffuse Reflectance Target, Edmund Optics) reference spectrum W(*λ*_*i*_) to obtain the normalised intensity spectra R(*λ*_*i*_) after subtracting the background spectrum B(*λ*_*i*_) from both as represented in Eq. (2)^33^.

2 $$R\left({\lambda }_{i}\right)=\frac{S\left({\lambda }_{i}\right)-B\left({\lambda }_{i}\right)}{W\left({\lambda }_{i}\right)-B\left({\lambda }_{i}\right)}$$

The reduced scattering coefficients (*µ*_*s*_*′*) and absorption coefficients (*µ*_*a*_) are then calculated using different scattering power coefficients (*b*), fractional chromophores (*f*_chromophore_), and their absorption coefficients in pure state (*µ*_*a,*chromophore_) using (*R*_pred_(*λ*_*i*_)) from the Monte-Carlo Look-Up Table (MCLUT) is obtained using these coefficients in the specified wavelength Eqs. (3) and (4), where *λ* is the wavelength range and *λ*_0_ is the reference wavelength^34–36^. The Look-Up Table (LUT) created with different absorption coefficients ranging from 0*.*0821 to 81*.*45 cm^*−*1^ and scattering coefficients (*µ*_*s*_) ranging from 1 to 60 cm^*−*1^, with the scattering anisotropy factor (*g*) of 0*.*85, created for earlier studies has been used here^32,34^. The tissue chromophores are iterated in the specified spectral ranges until the least square difference (*δ)* between them and the modeled reflectance spectra is minimal using Eq. (5)^37^. The fractional tissue chromophores water, lipid, Hb, and HbO_2_ for the least root mean square obtained gives an estimate of the functional information, including tissue moisture content, sO_2_, blood fraction, and lipid concentration. In this study, sO_2_ and blood fraction are calculated by Eqs. (6) and (7), respectively.

3 $${\mu }_{s}^{\mathrm{^{\prime}}}(\lambda )={\mu }_{s}^{\mathrm{^{\prime}}}\left({\lambda }_{0}\right){\left(\frac{\lambda }{{\lambda }_{0}}\right)}^{-b}$$

4 $${\mu }_{a}(\lambda )=\sum {f}_{\text{chromophore }}\cdot {\mu }_{a,\text{ chromophore }}(\lambda )$$

5 $$\delta =\sum_{i=0}^{k} {\left[R\left({\lambda }_{i}\right)-{R}_{\text{pred }}\left({\lambda }_{i}\right)\right] }^{2}$$

6 $$s{{\text{O}}}_{2}=\frac{{{\text{HbO}}}_{2}}{{{\text{HbO}}}_{2}+{\text{Hb}}}$$

7 $$\text{blood fraction }={f}_{{\text{Hb}}}+{f}_{{{\text{HbO}}}_{2}}$$

The average of these values for locations 1 to 6 were considered for the analysis, as these sites are symmetrically part of the vulva anatomy. To ensure easy comparisons, the values corresponding to each chromophore (water, lipid, sO_2_, blood fraction) were normalized using Python Scikit library’s MinMax scaler across the pre, peri, and postmenopausal groups as a unified population. This normalization process involved adjusting the values to a common scale (0 to 1), thereby transforming the raw data into relative values that highlight the proportional differences within and between groups. These normalized values are compared between the different categories of subjects to find trends relating to physiological changes. Mann Whitney U-test is performed on the results to obtain the p-value of the various locations, comparing the significance of their differences. To further validate the metrics obtained with DRS, they are compared with the currently accepted gold standard, VMI. The study subjects were dichotomised into low estrogen and normal estrogen based on the cut-off VMI score. Supervised classification between these groups using K-Nearest Neighbour (KNN) algorithm were performed^38^. The predictive model employed two datasets for training (75%) and testing (25%) with a fivefold cross validation to avoid over-fitting and ensure repeatability. The class labels were given based on the distance between the query point and nearest neighbours. The accuracy of classification was then calculated. The results with the average of locations 1 to 6 were compared with average of every two symmetric locations (1 and 4, 2 and 5, 3 and 6) as well as locations 7, 8, and 9, to advise on locations for the next study.

## Results

Table 1 summarizes the demographics, responses to the questionnaires, and clinical examination and measurements of the study cohort. Average BMI of the cohort was 23.6 kg/m^2^. The prevalence of vaginal dryness as the MBS impacted almost 50% (n = 31) of the peri and postmenopausal participants. In addition, VHI of postmenopausal women (3.54 ± 3.25) was lower than pre and perimenopausal women. pH measurements of the study cohort do not vary significantly between the three groups. The VMI measurements were 70.64 ± 13.10 for premenopausal, 57.83 ± 15.58 for perimenopausal, and 33.65 ± 31.54 for postmenopausal subjects (Table 1). Overall, 62% of the total subjects were currently sexually inactive and were ineligible to complete VQLI and VuHI as these include questions on current sexual activity. In the subgroup of currently sexually active women, the VQLI (4.09 ± 3.75) and VuHI (12.2 ± 1.99) scores in the postmenopausal women were higher compared to pre and perimenopausal women.

**Table 1:** Overview of participants’ demographics and profile based on: MBS, pH, VHI, VMI, VQLI, and VuHI in the study cohort (SD: standard deviation; n: number of subject(s)).

All subjects	Premenopausal (n = 35)	Perimenopausal (n = 15)	Postmenopausal (n = 50)
Age (years ± SD)	40 ± 9	47 ± 7	60 ± 6
Sexually active/past 4 weeks (n (%))	20 (57)	7 (46)	11 (22)
MBS			
Vaginal dryness (n (%))^a^	12 (34)	7 (46)	24 (48)
Discomfort (n (%))^a^	7 (20)	2 (13)	18 (36)
Dysuria (n (%))^a^	2 (5)	1 (6)	11 (22)
pH (mean ± SD)	5.16 ± 0.52	5.27 ± 0.50	5.90 ± 0.33
VHI (mean ± SD)	15.54 ± 3.32	13.33 ± 3.42	3.54 ± 3.25
VMI (mean ± SD)	70.64 ± 13.10	57.83 ± 15.58	33.65 ± 31.54

Sexually active subjects	Premenopausal (n = 20)	Perimenopausal (n = 7)	Postmenopausal (n = 11)
MBS			
Dyspareunia (n (%))^a^	6 (30)	3 (42)	3 (27)
Bleeding with sex (n (%))^a^	3 (15)	1 (14)	1 (9)
VQLI (mean ± SD)	1.4 ± 3.47	2.29 ± 2.43	4.09 ± 3.75
VuHI (mean ± SD)	1.35 ± 2.76	3.57 ± 3.20	12.2 ± 1.99

^a^Any scoring in the questionnaire other than “None” is considered as affected and are reported here).

**Figure 2:**
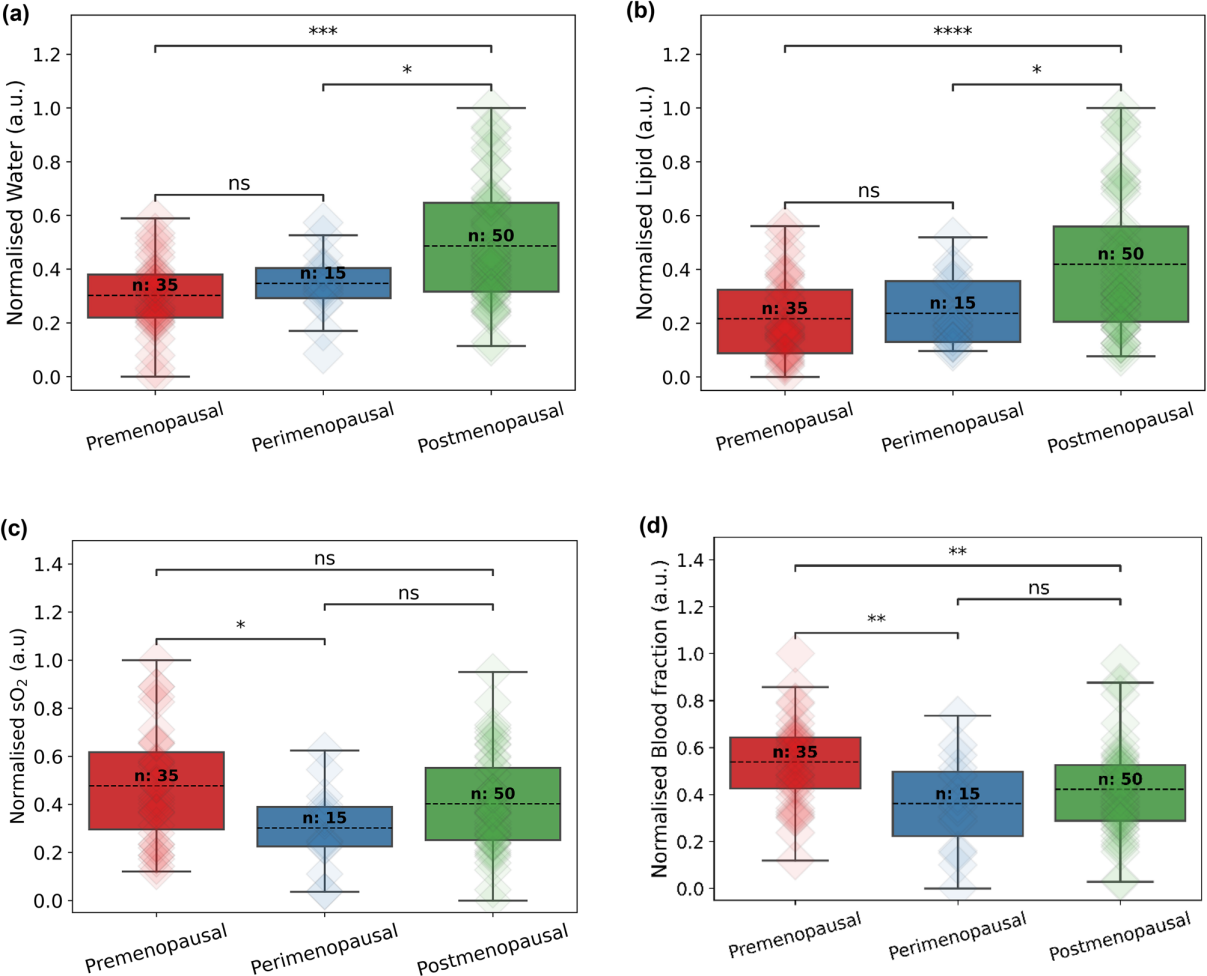
Normalised concentration of biochemical composition of vulva skin from the average of locations 1 to 6. (**a**) Water, (**b**) lipid, (**c**) sO_2_, (**d**) blood fraction. Two-sided Mann–Whitney U-test was performed yielding p-value of annotation legend: ns: non-significant, p > 0.05; *p < 0.05; **p < 0.01; ***p < 0.001; ****p < 0.0001.

For DRS analysis, all 100 patients were used as the measurements are not affected by the sexual activity of the participant. Figure 2 reports the relative amounts of water, lipid, sO_2_, and blood fraction obtained from the spectra. For water and lipid, there were no significant differences between pre and peri, a significant difference between peri and post (p < 0.05), and a more significant difference between pre and post (p < 0.001, p < 0.0001) as shown in Fig. 2a and b. The moisture content was observed to be higher in the postmenopausal participants, opposite to what was expected, while lipid followed the expected trend. In Fig. 2c, results show significantly less sO_2_ in peri compared to pre (p < 0.05). No significant difference was found comparing peri to post or pre to post. Finally, significant difference was found in blood fraction between pre and peri (p < 0.01) and pre and post (p < 0.01) while no significant difference was found comparing peri to post as shown in Fig. 2d. The captured high-resolution images of the vulva were not required as no abnormal spectra data was recorded.

**Figure 3:**
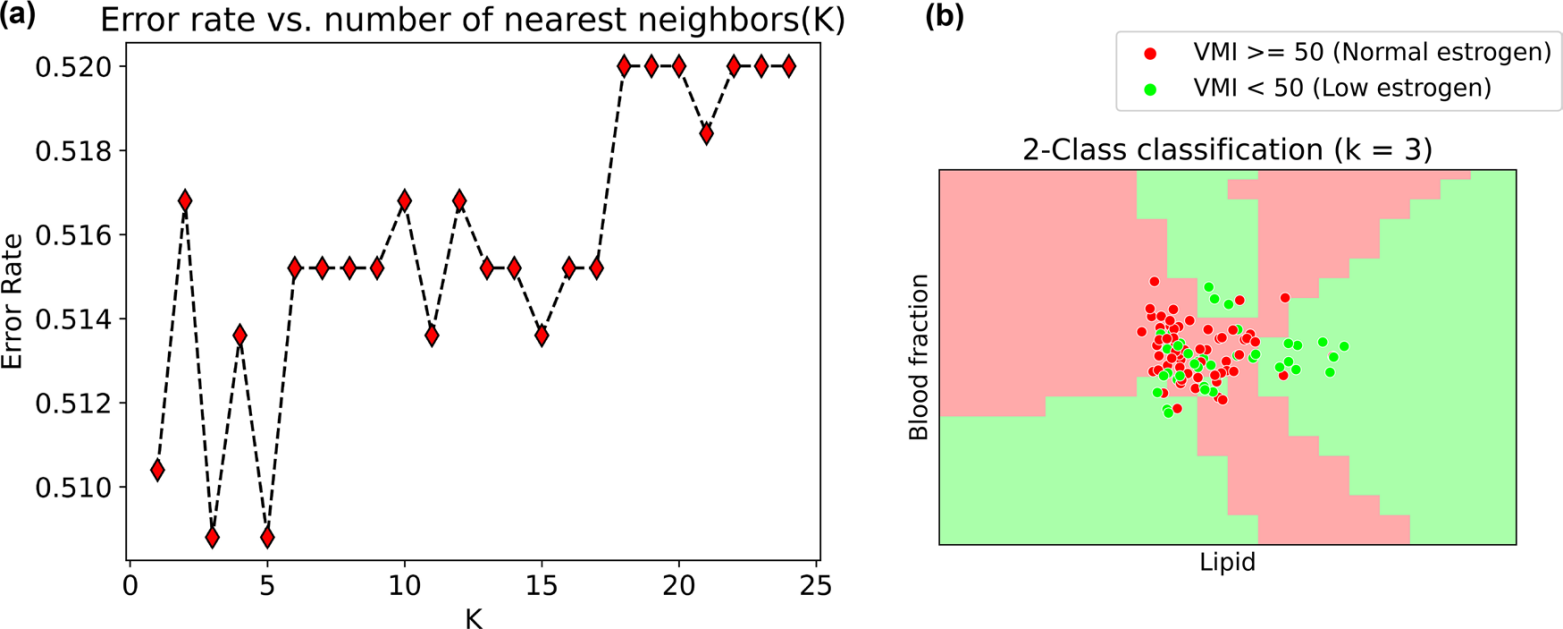
Supervised classification using KNN. (**a**) Representation of error rate varying with different nearest neighbour values. (**b**) KNN classification into low (red shaded) and normal (green shaded) estrogen categories with DRS lipid and blood fraction values in comparison with VMI score-based categories represented in the scatter plot.

As shown in Fig. 3a, the error rate is minimal for a nearest neighbour value of three which is used as input to the KNN classifier. The KNN classifier shows a prediction accuracy of 65% with VMI which indicates the estrogen level for an unbalanced dataset. The scatter plot shows the DRS tissue chromophore measurements of lipid and blood fraction in x and y axis. The overlap between the shaded region and dots of the same colour represents correct predictions as shown in Fig. 3b. Other DRS metrics like water, lipid, sO_2_, and blood fraction alone or in combination were less accurate (range 41–64%). Predictive accuracy was highest and equal for the combination of all four chromophores and that of lipid and blood fraction. Using two chromophores, compared to four, reduces the computation time hence, training with 2 features is employed here for the current probe. With the addition of a common clinical parameter, age, as a third training feature with the DRS chromophores, an accuracy of 78% was obtained. KNN classification results performed on age, existing subjective and objective measurements, and individual DRS tissue chromophores can be found in the supplementary information (Sect. 3, Table S1).

Table 2 compares all 4 tissue chromophores (i.e., water, lipid, sO_2_, and blood fraction) from the nine ROIs (Fig. 1b) to advise on the number of locations required for future study. The significance (*, **, ***, ****) noted in the Table 2 indicates a p-value less than 0.05 found for at least one of the tissue chromophores during the location comparison. We found no significant difference in the values of chromophores measured in the locations 1–6 and 8 suggesting similar chromophore distribution patterns. However, a significant difference was found at location 7 (mons pubis; p < 0.5) and location 9 (vaginal introitus, p < 0.0001). Regarding probe experience, 99% of participants rated the DRSI system as no discomfort or comfortable. The DRSI system was rated as easily performed in 98% of cases.

**Table 2:** Comparison of results from different probe locations to eliminate redundancy.

Tissue chromophores		P-value	Significance
Average 1 and 4	Average 2 and 5	> 0.05	ns
Average 3 and 6	Average 2 and 5	> 0.05	ns
Location 7	Average 2 and 5	< 0.05	*
Location 8	Average 2 and 5	> 0.05	ns
Location 9	Average 2 and 5	< 0.0001	****

## Discussion

This pilot study assessed the tissue chromophores extracted from the DRSI system and compared it to the estrogen status obtained using gold standard VMI score to investigate their use as surrogate markers for the non-invasive, objective assessment of GSM. Based on MBS, a higher proportion of peri/postmenopausal women were affected, out of which almost all the postmenopausal women had low VMI score, indicative of low estrogen effects.

The standard subjective indices VQLI and VuHI were collected in an attempt to validate the DRSI system performance. However, significant issues were identified with these indices leading to voided data, making comparisons challenging. The VQLI although validated in Australia^18^ for genital skin conditions, is very sexual function orientated and may be interpreted differently by Asians. The VuHI was compromised by the need for an experienced assessor and for the participant to report on symptoms during sexual intercourse. If one is not sexually active or less active, none would be scored, misleadingly elevating the overall score. This pragmatically limits its use in postmenopausal women as sexual activity is known to decrease with age^39^. This constituted 78% of postmenopausal women in our study. Based on the discrepancy in the number of subjects presented in DRS measurement and the subjective questionnaires, a direct comparison between them becomes unfeasible. The limited overlap in subjects between the datasets, prevents meaningful comparative analysis. Consequently, the datasets cannot be reliably compared due to the inherent disparity in subject numbers and hence, were disregarded from further analysis. Vaginal pH is a validated objective assessment tool which has been previously reported but it requires an invasive examination^40^. Little is known about the vaginal microbiome in Asian women throughout the life course, with only small studies published^41^. The VHI is invasive and requires both pH and an experienced assessor. Davila et al. demonstrated only moderate correlation between VHI and VMI^41^.

Regarding the tissue chromophores signatures, previous work found significant variations in lipids obtained from tissue spectra between pre and postmenopausal women^42–44^. However, the DRSI system overcomes the bulkiness of a typical Raman probe and measures functional information. For water, we expected the reverse of our results as postmenopausal skin is dry as the estrogen deficiency may reduce the attraction of water molecules as studied with the cervical tissue^45–47^, and in an overictomised mice, where the water reduction is caused due to epidermal barrier dysfunction^48^. A possible explanation for this result is that due to epidermal thinning, in response to estrogen deprivation with age^49^, the investigating depth of the probe with SDS 2.7 mm was deeper than required. The results for subjects in the peri and postmenopausal stage may therefore reflect the dermis rather than the epidermis. In contrast, the higher lipid content found in our postmenopausal women has also been reported in the cervix^41^. This is because estrogen improves the vaginal blood flow and reduces the level of low-density lipoprotein (LDL) through enhancing the LDL receptor activity level which in turn increases the fractional catabolic rate for LDL^41^, which was observed in fasting blood stream earlier^50–52^. Finally, compared to premenopausal skin, the haemoglobin absorbance in postmenopausal women was reported to be lower by Farage et al. using infrared spectroscopy^53,54^. This was reported to be due to decreased vasculature with aging. This is consistent with the reduction in sO_2_, and blood fraction observed in postmenopausal women with our system. The results with KNN classifier showed that the highest accuracy of the spectra evaluated by the DRSI was a combination of lipid and blood fraction with a prediction accuracy of 65% as compared to VMI. The addition of clinical parameter such as age as a third training feature to the model increased the accuracy to 78%. However, the primary focus of this study is to validate the DRSI derived tissue chromophores which can be potentially improved with the addition of water chromophore by measuring it in the superficial epidermis with an optimised fiber probe.

In terms of the number of locations of interest that need to be measured, locations 1–6 and 8 were similar, suggesting that minimal locations could be measured and still be representative of the entire vulva (Table 2). This would reduce measurement time and make the device easier for non-experts. The mons area (location 7) gave a different spectral signature as expected anatomically, due to it not being a part of the genitourinary tract. Although the introitus (location 9) is recognized as a crucial site for genital sensitivity, the notably distinct result indicates that this area may not be suitable for a flat probe system. The high-resolution vulva images captured were used for the initial inspection of the data quality to record any anatomical changes and for reviewing abnormal spectra. These images can also be used for treatment monitoring and visual examination of the dryness in the future clinical adaptation of our system. This has been demonstrated by Irwin et al. with vulvar vestibular photography^55^.

One of the study’s limitations include its exclusive focus on fair skin, warranting further investigation for application to darker skin tones. The effect of hair in the vulva area on readings remains uncertain. Another considerable factor is the tissue properties used for the MCLUT simulation as this was referred from a standard epithelial tissue and may vary for the vulva tissue. This is also accompanied by the standard limitations to the accuracy of Monte Carlo simulation for skin optical properties^56^. VMI, as a gold standard, risks interobserver variability in the calculation of the maturation value because only 100 cells are counted. In contrast, strengths include the following: robust categorisation to pre, peri, and postmenopausal; assessment of multiple areas of interest; ease of use and acceptability; and direct comparison to a gold standard while discussing the disadvantages of the existing subjective and objective assessment tools. Future research directions include investigating the impact of different probe depths on water chromophore measurements, assessing the individual and combined accuracy of the assessment method with VMI, and exploring changes in assessment results with vaginal estrogen treatment.

## Conclusion

In this study, we present a DRSI system to deduce tissue chromophores in the vulva skin as surrogate markers, to assess GSM. The proposed tool is non-invasive, quick, and acceptable to both patients and clinicians. We observed quantitative changes in key tissue parameters such as water, lipid, sO_2_, and total blood fraction between pre, peri and postmenopausal subjects. We identified changes in lipid, sO_2_, and blood fraction in the vulva skin that may serve as an objective biomarker to assess GSM symptoms. Moreover, when lipid and blood fraction indices derived from the DRS were used in a KNN classifier model, a prediction accuracy of 65% was achieved as compared to VMI, which is the clinical research tool to assess estrogen-related hormonal changes. By incorporating age as an additional training feature, the model achieved an accuracy of 78%. Currently, we are developing a second-generation DRS probe that can interrogate the epidermal layer of vulva skin in postmenopausal subjects. With further refinement of the study protocol and DRS probe configuration to interrogate the superficial/deeper vulva skin, DRSI has the potential to objectify the diagnosis and management of GSM. Incorporation of Raman spectroscopy into the probe will offer a quantitative assessment of estrogen content in the vulva skin. We envision that such a device could potentially fit into the routine clinical workflow and will be a promising objective assessment tool for point of care diagnosis. It could also aid in monitoring the response to treatment for GSM and other vulva lesions.

### Supplementary Information

Supplementary Information.

## Data Availability

Data is available upon request and should be addressed to USD, SL, or MO.
