# Supplementary material for: Diffuse reflectance spectroscopy and imaging for non-invasive objective assessment of genitourinary syndrome of menopause: a pilot study
Source: Sci Rep. 2024 Jan 11;14:1085. doi: 10.1038/s41598-023-49655-4 (PMC10784538; doi:10.1038/s41598-023-49655-4)
Supplement: Supplementary file 1 — Supplementary Information. [file 41598_2023_49655_MOESM1_ESM.docx]

Diffuse reflectance spectroscopy and imaging for non-invasive objective assessment of genitourinary syndrome of menopause - A pilot study.

# U.S. Dinish1,2,*,+, Susan Logan3,4,5,*,+, Ghayathri Balasundaram1,2,+, Valerie Teo Xinhui1,2, Keertana Vinod Ram1,2, Zhang Ruochong1,2, Bi Renzhe1,2, Steffie Silvani5, Kee Hua Cheng6, Xu Xia6, Goh Giap Hean6, Mahesh Choolani3,5, and Malini Olivo1,2,*

1Institute of Materials Research and Engineering (IMRE), Agency for Science, Technology and Research (A*STAR), 2 Fusionopolis Way, Innovis #08-03, Singapore 138634, Republic of Singapore 2A*STAR Skin Research Labs (A*SRL), Agency for Science, Technology and Research (A*STAR), 31 Biopolis Way, #07-01 Nanos, Singapore 138669, Republic of Singapore

3Department of Obstetrics and Gynaecology, NUS Yong Loo Lin School of Medicine, National University of Singapore, 1E Kent Ridge Road, NUHS Tower Block, Level 12, Singapore 119228, Singapore.

4Department of Sexual and Reproductive Health, NHS Grampian, Scotland

5Department of Obstetrics and Gynaecology, National University Hospital, 1E Kent Ridge Road, NUHS Tower Block, Level 12, Singapore 119228, Singapore.

6Department of Pathology, National University Hospital, 5 Lower Kent Ridge Road, Singapore 119074

**2,4^:^ current affiliation**

+Joint first authors

***Corresponding authors:** dinish@asrl.a-star.edu.sg; susan_logan@nuhs.edu.sg; (current) susanlogan@nhs.scot; malini_olivo@asrl.a-star.edu.sg

**Supplementary Information**

1. **Questionnaires:**

# Participant’s Questionnaire (complete by participant)

**Most Bothersome Symptoms (please tick)**

|  | None | Mild | Moderate | Severe | Not  applicable |
| --- | --- | --- | --- | --- | --- |
| Vaginal dryness |  |  |  |  |  |
| Dyspareunia |  |  |  |  |  |
| Irritation/burning/itching |  |  |  |  |  |
| Dysuria |  |  |  |  |  |
| Bleeding with sex |  |  |  |  |  |

Reference: Ettinger B et al., Menopause 2008 15(5) 885-889

# The Vulvar Quality of Life Index (VQLI) questions

1. Over the past month how itchy and/or painful and/or stinging and/or burning has your vulvar skin felt?


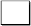

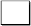
Very much A lot


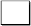

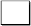
A Little

Not at all

1. Over the past month, how often have you experienced any of the following: pain when urinating, heat intolerance, vaginal discharge, wetness?


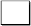

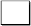
Very much A lot


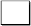

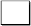
A Little

Not at all

1. Over the last month how embarrassed or self-conscious have you been because of your vulvar skin symptoms?


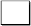

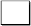
Very much A lot


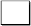

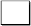
A Little

Not at all

1. Over the past month how much has your vulvar skin impacted your body image or sense of self? (For instance, sense of femininity, feeling isolated, feeling different)?


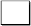

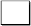
Very much A lot


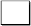

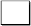
A Little

Not at all

1.
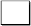
Over the last month how distressed or anxious have you felt because of your vulvar skin problem? Very much


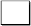
A lot


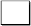

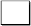
A Little

Not at all

1. Over the last month how much has your vulvar skin problem influenced your choice of clothing (For instance underwear, jeans, gym clothes)?


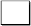

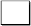
Very much A lot


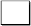

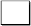
A Little

Not at all

1.
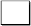
Over the last month how much has your vulvar skin problem disturbed your sleep? Very much


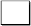
A lot


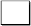

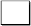
A Little

Not at all

1. Over the last month how much has your vulvar skin problem made it difficult for you to go shopping, look after yourself or your family, home and garden?


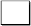

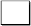
Very much A lot


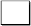

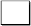
A Little

Not at all

1. Over the last month how much has your vulvar skin problem made it difficult for you to attend social or leisure engagements? (For instance, going out for dinner or bars, dating, sport, exercise class, gym)


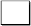

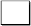
Very much A lot


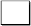
A Little


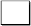
Not at all

1. Over the last month how much has your vulvar skin problem interfered with your ability to concentrate on work or study?


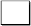

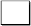
Very much A lot


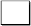
A Little


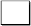
Not at all / Not applicable

1. Over the last month how much has your vulvar skin problem created problems with a partner or precluded you from pursuing a romantic relationship? (For instance, maintaining a relationship or finding a partner)


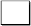

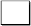
Very much A lot


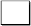
A Little


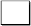
Not at all / Not applicable

1. Over the last month how much has your vulvar skin problem interfered with your sex life? (For instance, decreased libido, decreased frequency of sex, pain with sex and/or enjoyment of sex)


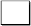

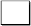
Very much A lot


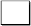
A Little


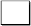
Not at all /Not applicable

1. Over the last month how often have you felt distressed or worried about sex because of your vulvar skin?


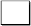

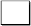
Very much A lot


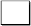
A Little


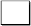
Not at all / Not applicable

1. How often in the last month have you been worried about long-term health implications of your vulvar skin condition? (For instance, concern about developing cancer or difficulties with fertility)


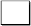

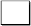
Very much A lot


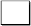

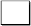
A Little Not at all

1. Over the past month, how much of a problem has the treatment of your vulvar symptoms been

(For instance messy, time consuming, expensive, inconvenient)?


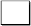

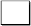
Very much A lot


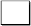
A Little


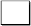
Not at all / Not applicable

| **Overall VQLI Score Range** | **Impact on Quality of Life** |
| --- | --- |
| 0 to 5 | minimal |
| 6 to 13 | mild |
| 14 to 23 | moderate |
| 24 to 37 | severe |
| 38 to 45 | very severe |

Reference: Saunderson RB et al. Vulvar quality of life index (VQLI) - A simple tool to measure quality of life in patients with vulvar disease. Australas J Dermatol. 2020 May; 61(2):152-157

# Clinical examination and measurements (complete by PI/Co-I)

**Vulval Health Index***

A score of 3 in any given category or a total score of >8 suggests the presence of severe vulvar atrophy

| Please circle | Normal  0 | Mild  1 | Moderate  2 | Severe  3 |
| --- | --- | --- | --- | --- |
| Labia majora | Normal | Mild loss | Moderate loss | Severe loss or  disappeared |
| Labia minora | Normal | Mild loss | Moderate loss | Severe loss or  disappeared |
| Clitoris | Normal size | Mild decrease in size | Moderate decrease in  size | Severe loss or undetected |
| Introitus & elasticity | Normal | Mild decrease or stenosis | Moderate decrease or  stenosis | Severe decrease or stenosis |
| Colour | Normal | Mild pallor | Moderate  pallor | Severe pallor |
| Discomfort and pain | Normal | Mild during intercourse | Moderate during intercourse | Severe during intercourse and any discomfort intensity beyond  intercourse |
| Other findings eg petechiae, excoriation,  ulceration | Normal | Mild | Moderate | Severe |

Reference: Palacios S et al. Vulvar and vaginal atrophy as viewed by the Spanish REVIVE participants: symptoms, management, and treatment perceptions. Climacteric 2017; 20: 55–61

**Vaginal pH**

Two inch paper grasped by sponge forceps and placed against the middle to upper 1/3^rd^ of the lateral wall of the vagina (N 3.8-4.2)

PH:

**Vaginal Health Index***

|  | Please circle | | | | |
| --- | --- | --- | --- | --- | --- |
| Overall elasticity | None | Poor | Fair | Good | Excellent |
| Fluid secretion type and consistency | None | Scant, thin yellow | Superficial, thin white | Moderate, thin white | Normal, white  flocculent |
| pH | 6.1 | 5.6-6.0 | 5.1-5.5 | 4.7-5.0 | < 4.6 |
| Epithelial mucosa | Petechiae noted  before contact | Bleeds with light contact | Bleeds with scraping | Not friable, thin mucosa | Not friable,  normal mucosa |
| Moisture | None, mucosa inflamed | None, mucosa not  inflamed | Minimal | Moderate | Normal |

Reference: Bachmann G. Urogenital ageing: an old problem newly recognized. Maturitas 1995; 22:S1–S5

Lower score corresponds to greater urogenital atrophy; range 5-25; If the total score is < 15 the vagina is atrophic

**Vaginal Maturation Index***

The Vaginal Maturation Index (MI) quantifies the estrogen status of the vaginal epithelium. Three epithelial cell types, parabasal, intermediate and superficial, are used to evaluate vaginal estrogenization. A predominance of parabasal cells (the least mature type) and absence of superficial cells indicates a low concentration of circulating estrogens (free estradiol, estrone, and estriol). A predominance of superficial epithelial cells (the most mature type) indicates higher concentrations of circulating estrogens. Intermediate cells are moderately mature.

| Please circle | Parabasal | Intermediate | Superficial |
| --- | --- | --- | --- |
| VMI (%) |  |  |  |
| Circulating Estrogen | Low | Moderate | High |

Reference: Lindau ST, Dude A, Gavrilova N, Hoffmann JN, Schumm LP, McClintock MK. Prevalence and correlates of vaginal estrogenization in postmenopausal women in the United States.

Menopause. 2017;24(5):536-545. doi:10.1097/GME.0000000000000787

1. **Thermal load in vulva skin**

To determine the thermal load in vulva skin, we employ the specific heat capacity formula as shown in Equation 1.

$\Delta T=\frac{Q}{mc}$ (1)

$\Delta T=\frac{Output power \times exposure time}{mc}$

$\Delta T=\frac{(4.5\times{10}^{-3})W \times30s}{1g \times2.98 J/g^{\circ}C} =0.0453^{\circ}C$

Q = mcΔT where Q is energy, m is the mass of the tissue, c is the specific heat capacity of a typical biological tissue, and ΔT is the change in temperature of the vulva skin.

The probing area of the vulva skin at a single site is approximately 8.48 mm², based on the size of the probe. As a result, the region being examined is quite small, and we estimate its maximum mass to be lower than 1 g. In our calculations, we assume the mass of the vulva probing region to be around 1 g (considering it is the maximum possible value) and utilize a specific heat capacity of 2.98 J/g°C for biological tissues^1^. Consequently, the overall temperature increase in the vulva skin is estimated to be approximately 0.0453 °C, which is practically negligible and poses no concern in our application.

Additionally, the light source we use is white light (broadband), which is not a coherent light source. This characteristic further minimizes the thermal load, making it even more inconsequential.

1. **KNN classification results**

| **Statistical Analysis - KNN classification (n=100)** | |
| --- | --- |
|  | **Accuracy with VMI** |
| **Clinical Parameter** | |
| Age | 76% |
| **Subjective Measurements** | |
| Vaginal Health Index (VHI) | 80% |
| **Objective Measurements** | |
| pH | 81% |
| **DRSI Measurements** | |
| Water | 58% |
| Lipid | 56% |
| sO_2_ | 41% |
| Blood Fraction | 49% |
| Combination (Water, Lipid, sO2, Blood fraction) | 50% |
| Combination (Lipid, Blood fraction) | 65% |

**Table S1.** Accuracy for KNN classification of clinical parameters, different subjective measures and DRSI tissue chromophore measurements into low and normal estrogen categories as compared to the gold standard VMI.

**References**

1. Xu, Xiaojiang & Rioux, Timothy & Castellani, Michael. (2022). The specific heat of the human body is lower than previously believed: The Journal Temperature toolbox. Temperature. 10. 1-5. 10.1080/23328940.2022.2088034.
